# Moving towards universal coverage in South Africa? Lessons from a voluntary government insurance scheme

**DOI:** 10.3402/gha.v6i0.19253

**Published:** 2013-01-24

**Authors:** Veloshnee Govender, Matthew F. Chersich, Bronwyn Harris, Olufunke Alaba, John E. Ataguba, Nonhlanhla Nxumalo, Jane Goudge

**Affiliations:** 1 Health Economics Unit, School of Public Health and Family Medicine, Faculty of Health Sciences, University of Cape Town, South Africa; 2 Centre for Health Policy, School of Public Health, Faculty of Health Sciences, University of the Witwatersrand, Johannesburg, South Africa; 3 Department of Obstetrics and Gynaecology, International Centre for Reproductive Health, Ghent University, Ghent, Belgium

**Keywords:** health insurance, civil servants, health-finance reforms, universal coverage, South Africa

## Abstract

**Background:**

In 2005, the South African government introduced a voluntary, subsidised health insurance scheme for civil servants. In light of the global emphasis on universal coverage, empirical evidence is needed to understand the relationship between new health financing strategies and health care access thereby improving global understanding of these issues.

**Objectives:**

This study analysed coverage of the South African government health insurance scheme, the population groups with low uptake, and the individual-level factors, as well as characteristics of the scheme, that influenced enrolment.

**Methods:**

Multi-stage random sampling was used to select 1,329 civil servants from the health and education sectors in four of South Africa's nine provinces. They were interviewed to determine factors associated with enrolment in the scheme. The analysis included both descriptive statistics and multivariate logistic regression.

**Results:**

Notwithstanding the availability of a non-contributory option within the insurance scheme and access to privately-provided primary care, a considerable portion of socio-economically vulnerable groups remained uninsured (57.7% of the lowest salary category). Non-insurance was highest among men, black African or coloured ethnic groups, less educated and lower-income employees, and those living in informal-housing. The relatively poor uptake of the contributory and non-contributory insurance options was mostly attributed to insufficient information, perceived administrative challenges of taking up membership, and payment costs.

**Conclusion:**

Barriers to enrolment include insufficient information, unaffordability of payments and perceived administrative complexity. Achieving universal coverage requires good physical access to service providers and appropriate benefit options within pre-payment health financing mechanisms.

In 2005, member states of the World Health Organization (WHO) committed themselves to developing health financing systems that would enable universal coverage (UC) by ensuring access to adequate health care at an affordable cost for all citizens (1). Although there is no one clear path to UC, the World Health Report 2010 describes several strategies for expanding access to care. These include the removal of direct payments, particularly user fees, and the introduction of pre-payment schemes with tax-based funding or compulsory or voluntary health insurance contributions (2).

Given the limits of, and competing demands on, tax-based funding (3), the focus in many low- and middle-income countries, has been on contributory health insurance schemes (where employees contribute toward the premium). Nonetheless, there is the recognition that for some groups, these contributions will need to be partially- or fully-subsidised by government (2). Some countries such as Iran are expanding coverage through voluntary, contributory insurance schemes (4), while Ghana is opting for mandatory insurance (5). Countries with existing insurance schemes, have attempted to expand coverage through the provision of lower cost alternatives with similar benefit packages, but possibly with limited choice of providers. The Seguro Popular Programme in Mexico and the UC scheme in Thailand, are examples where the contributions of low-income individuals and families are subsided by government (6, 7).

In South Africa the tax-funded public health system, with free primary health care and minimal charges for inpatient care, provides some form of UC. However, despite substantial transformation of the public health system post-apartheid (8, 9), perceptions and experiences of poor quality of public health care persist (10). These arise from a range of factors, including the quadruple disease burden (11), poor stewardship, and inefficient use of resources (8). This has lead to increased utilisation of private providers for primary health care. However, only the wealthiest 16% of the population can afford private health insurance to cover the costs of private-sector services (12). For the uninsured, direct payments are often catastrophic in nature (above 10% of household expenditure) (13), contributing to household poverty (14, 15). Therefore, despite a tax-funded public health care system available to all, marked inequalities in health care access persist (8, 16).

It is against this backdrop that the goal of UC has taken centre stage in several health-financing reform policy proposals and initiatives since democracy in 1994. Box 1 shows a timeline of policy initiatives and proposals (17–26).

***Box 1*. Timeline of health-financing policy initiatives and proposals since 1994**

**1994**: African National Congress (ANC) National Health Plan recommended that a Commission of Inquiry be appointed to investigate the feasibility of a National Health Insurance (NHI) Fund (17).

**1994:** National Department of Health's Health Care Finance Committee put forward three possible mandatory insurance options, including NHI (18).

**1995:** Hospital Strategy Project, initiated by the National Ministry, tasked with setting out a framework for the development and restructuring of the public hospital sector (19).

**1995:** Committee of Inquiry into a NHI System (20).

**1997:** National Department of Health releases policy document on Social Health Insurance Scheme for formal sector employees (21).

**1997:** White Paper on the transformation of the health system in South Africa built upon the ANC's 1994 Health Plan (22).

**2002**: The Committee of Inquiry into a Comprehensive System of Social Security for South Africa recommends that South Africa move toward a NHI system (23).

**2004**: Ministerial Task Team on SHI recommended implementation of SHI for the formally employed, since it did not consider NHI feasible in the short term.

**2005**: Ministerial Task Team commissioned an investigation into low-income medical schemes (24).

**2005**: Introduction of the Government Employee Medical Scheme (referred to as the government scheme in the article), restricted to public-sector employees.

**2007**: A policy resolution committed the ANC to introduce NHI (25).

**2011:** NHI Green Paper released by government detailing a 14-year plan towards NHI (26).

As indicated in these timelines, the earlier debates considered the option of a NHI scheme which, by definition ‘covers the entire population irrespective of whether they have personally contributed to the scheme or not’ (12, p. 73). Around 2005 strategies for insurance coverage of low-income households were considered in the country (24). In 2005, the government (as an employer) implemented a health insurance scheme (27), restricted to government employees, that aimed to achieve greater pooling of funds across this segment of the employed population. Post 2009 the debate has shifted to the implementation of a NHI system, that aims to strengthen the public health care system and ensure adequate provision of funding (26).

South Africa's government employees’ scheme intends to pool resources from a broad range of civil servants and aims to attract members from all income groups. The intention of a designated network of private general practitioners and private hospitals is to expand access to benefits for low-income government employees. The scheme is heavily subsidised, particularly for low-income members, to encourage enrolment and so extend coverage.^1^ Employees appointed from 1 July 2006 onwards were only eligible for the government subsidy if they joined the government scheme and not another health insurance scheme.^2^

This study analysed coverage of the government health insurance scheme, the population groups with low uptake, and the individual-level factors, as well as characteristics of the scheme that influenced enrolment.

The study findings are used to highlight lessons for other contributory schemes which aim to encompass both high- and low-income population groups.

## Methods

### Sampling and data collection

In 2008–09, 1,329 currently employed civil servants were interviewed across four of South Africa's nine provinces.^3^ Health and education, two of the largest public sectors, were selected for the survey. Provinces were chosen on the basis of being urban, having a greater distribution of private providers as well as relatively well-resourced public health care facilities (Gauteng and Western Cape), and being predominantly rural with few private facilities and less-resourced public facilities (KwaZulu-Natal and North West) in order to assess variation in enrolment related to geographical access. The minimum sample size per province was 245 and this was increased to 309 to allow for possible incomplete questionnaires.

Multi-stage random sampling was used. First, the number of health and education employees to be sampled in each salary category was determined by their relative proportion in each province. Second, districts in each province were selected with a probability proportionate to number of employees, following which 15 schools and four hospitals within each of the selected districts were randomly selected. Finally, within the selected schools and hospitals, a sampling frame was constructed of all employees, stratified by salary category, to allow specific quotas of interviews to be conducted across the different salary categories. These employees were then invited for an interview until the required number in each salary category was reached. Study procedures received ethics clearance from the Universities of Cape Town and the Witwatersrand, as well as relevant Provincial Departments of Health. All respondents provided informed signed consent.

### Study variables and data management

Information was collected on health insurance uptake, including membership of the government and other schemes, factors influencing membership of the government scheme, choice of benefit option and the reasons for such choice. Those who transferred to the government scheme from another medical scheme were classified as ‘previously-insured’, while ‘newly-insured’ referred to those who were uninsured prior to joining the government scheme. The survey questionnaire included questions to assess possible consumer inertia, arising from the transaction costs of either switching from one scheme to another (28) or from joining a scheme having not been previously insured. The questionnaire also included reasons underlying inertia, specifically the lack of a perceived need for insurance and administrative complexity of the scheme.

Adverse selection, arising from the tendency for people with perceived low risk (younger, healthier, low-income) to avoid insurance coverage is another challenge for voluntary schemes (29). In recognition of this, age, health status and income (indicated by salary) were assessed as key potential determinants of the decision to take insurance. The choice of socio-demographic (age, sex, race, location, education, marital status and housing), economic (income) and health status variables was guided by previous research examining determinants of health insurance ownership in voluntary schemes in South Africa (30) and internationally (31–34).^4^ Civil servants were classified by skill level into five categories (lower skilled, skilled, highly-skilled, supervisory and senior management); these categories determine salary levels within the civil service.

Data were double-entered by an independent survey company, cross-checked by the research team and then analysed using Stata^®^ 10 (Stata Corporation, College Station, TX, United States). In addition to descriptive statistics of the uninsured and insured populations, the respondent's decision to enrol in the government scheme was modelled using multivariate logistic regression. The dichotomous dependent variable was enrolment in the government scheme (combining the ‘previously-insured’ and ‘newly-insured’) and the explanatory variables were categorical and included socio-demographics, salary level and health status. Variables associated with government scheme membership in univariate analysis (*p*<0.1) were included in the initial multivariate model in addition to important potential confounders such as gender, and retained if their removal markedly altered the model fit. Education level was excluded as one of the independent variables since it correlated closely with salary level.

## Results

### Description of study population

Two-thirds of respondents worked in the education sector, and one third in health. More than half (58.6%) were female. A third of respondents were 30–39 years and a similar proportion was 40–49 years (35.2%). Approximately two-thirds had tertiary-level education, while 12.5% had only primary or no education. Median total monthly household expenditure was US$533.3.^5^ Half the respondents were classified as highly skilled employees (53.3%); almost a third (31.1%) as being low skilled; and only 3.1% were senior managers. Only 2.6% reported their health as being poor or very poor, but almost a third was taking chronic medication.

### Insurance status of public-sector employees

Three-quarters of respondents (74.3%) were insured (with either the government or another insurance scheme Table 1) and 25.7% are uninsured. Less than half (41.9%) of the insured are members of the government scheme; more than half of the insured (58.1%) belonged to another scheme. Of the members of the government scheme, 29.5% had insurance prior to joining the government scheme, while 12.4% were newly-insured. Of the 9.2% of the respondents who joined the civil service after 2006, only 18.9% had enrolled in the government scheme. A further 16.4% were members of another scheme, considerably fewer than amongst civil servants employed before 2006 where almost half were enrolled in another scheme (46.0%); the remainder (64.7%) were uninsured.

**Table 1:** Associations between health insurance, demographic characteristics and income level among public-sector employees in South Africa

		Insurance scheme (%)			
			
		Government scheme			
				
Variable (n)	Insured% (n)	Newly insured	Previously insured	Other schemes	*p*
Age (years)					
20–29 (141)	53.9 (76)	39.5	19.7	40.8	<0.001
30–39 (402)	74.4 (299)	18.7	26.4	54.9	
40–49 (468)	78.6 (368)	6.3	31.3	62.5	
50–59 (268)	76.5 (205)	5.9	35.1	59.0	
≥60 (46)	80.5 (37)	2.7	27.0	70.3	
Sex					
Female (778)	78.8 (613)	13.4	28.2	58.4	0.317
Male (548)	67.7 (371)	10.8	31.8	57.4	
Marital status					
Married/cohabiting (806)	77.8 (627)	7.3	30.6	62.1	<0.001
Div./sep./widow (149)	77.2 (115)	8.7	38.3	53.0	
Single (375)	65.6 (246)	26.8	22.8	50.4	
Education level					
None/prim. comp (168)	48.8 (82)	18.3	30.5	51.2	<0.001
Incomp. secondary (102)	66.7 (68)	14.7	33.8	51.5	
Comp. secondary (184)	63.0 (116)	23.3	39.7	37.0	
Diploma (360)	79.2 (285)	14.7	27.7	57.6	
Degree (516)	84.7 (437)	6.4	27.2	66.4	
Housing					
Formal (1272)	75.9 (966)	11.8	29.8	58.4	0.003
Informal (50)	40.0 (20)	40.0	20.0	40.0	
Race					
Black African (858)	71.1 (610)	16.1	27.9	56.0	<0.001
Coloured (253)	70.7 (179)	10.0	33.0	57.0	
Indian/Asian (77)	87.0 (67)	6.0	34.3	59.7	
White (132)	93.9 (124)	0.8	31.5	67.7	
Salary category					
Lower skilled (168)	42.3 (71)	31.0	22.5	46.5	<0.001
Skilled (246)	60.6 (149)	27.5	37.6	34.9	
Highly-skilled (709)	81.2 (576)	9.6	28.3	62.1	
Supervisory and Senior Management (206)	92.7 (191)	2.1	29.8	68.1	
Province					
Gauteng (344)	70.4 (242)	17.7	32.3	50.0	<0.001
KwaZulu-Natal (310)	72.6 (225)	17.3	24.9	57.8
North West (329)	82.4 (271)	5.9	29.2	64.9
Western Cape (343)	72.6 (249)	9.6	31.8	58.6
Self-assessed health status					
Excellent (320)	71.3 (228)	17.6	29.8	52.6	0.021
Good (633)	75.8 (480)	10.2	31.0	58.8	
Average (342)	74.5 (255)	11.0	25.5	63.5	
Poor (34)	73.5 (25)	20.0	40.0	40.0	
Individual on chronic medication					
Yes (385)	86.7 (334)	8.1	32.3	59.6	0.013
No (923)	69.5 (641)	14.4	27.9	57.7	
Total	74.3 (988)	12.4	29.5	58.1

The insured (those belonging to the government or other schemes) were more likely to be above 40 years, women, educated at tertiary level, living in formal housing, Indian/Asian or white, in the higher salary categories (highly skilled to senior management) and living in a household with an individual on chronic medication. Self-assessed health status was not a predictor of health insurance. In univariate analysis, all socio-economic and demographic variables, besides gender, were associated with uptake of the government scheme (either newly or previously insured).

Taking up insurance for the first time (newly insured) was highest amongst those aged 20–29, females, single people, black Africans, and those living in informal housing or with a lower-income (salary categories lower skilled and skilled) (Table 1). In contrast, factors associated with switching from a previous scheme to the government scheme were having a skilled job, age 50–59 years, being divorced, separated or widowed, secondary education level, living in formal housing and in an urban province (i.e. Gauteng or the Western Cape).

Multivariate analysis allowed for the simultaneous examination of the effect of several demographic, socio-economic and health status factors on the uptake of the government insurance scheme by both the previously and newly insured (Table 2). Multivariate analysis showed that employees who were female, no longer married or cohabiting (i.e. divorced, separated, widowed), or in the lowest salary category were more likely to have enrolled in the government scheme (Table 2). Enrolment in the government scheme was 72% lower among those >60 years and 43% lower in those 40–49 years, compared with those aged 20–29 years. Similarly, those living in the relatively rural provinces (KwaZulu-Natal and North West) were less likely to be insured under the government scheme than the urban provinces.

**Table 2:** Multivariate logistic regression analysis of factors associated with uptake of the government insurance scheme (newly insured and previously insured by other schemes)

	Adjusted odds ratio	(95% CI)
Age	
20–29	1.0	
30–39	0.83	0.47–1.48
40–49	0.57*	0.32–1.03
50–59	0.62	0.33–1.17
≥60	0.28*	0.11–0.74
Sex		
Female	1.0	
Male	0.69*	0.51–0.93
Marital status		
Married/cohabiting	1.0	
Divorced/separated/widowed	1.60*	1.03–2.48
Single	1.35	0.97–1.90
Housing		
Formal	1.0	
Informal	1.73	0.63–4.72
Salary category		
Lower skilled	1.0	
Skilled	1.51	0.82–2.75
Highly-skilled	0.48**	0.28–0.81
Supervisory and Senior Management	0.39**	0.22–0.71
Province		
Gauteng	1.0	
KwaZulu-Natal	0.69*	0.46–1.01
North West	0.58**	0.40–0.85
Western Cape	0.76	0.52–1.11
Individual on chronic medication		
Yes	1.0	
No	0.94	0.69–1.27

CI, confidence interval.

* *p*<0.1

** *p*<0.05.

### Choice of benefit option under the government scheme

The government scheme has five benefit packages ranging from low-cost options, which are fully subsidised for those in the lower income categories, to high-cost packages that are increasingly comprehensive in the range of services covered. The two lower-cost options (options 1 and 2) offer members outpatient benefits through a limited network of private healthcare providers (general practitioners, dentists or optometrists). These two options differ with respect to hospital benefits; in option 1, members have access to a network of state hospitals and option 2 to a limited private hospital network.^6^ Options 3, 4 and 5 allow access to any private hospital.

Option 4 was the most popular benefit option, with the proportion selecting this option rising as salary increased (Table 3). However, a substantive proportion (28.3%) of those in the highest salary category selected comprehensive option 5. Of the two low-cost options, the fully subsidised option 1 was more popular amongst the lowest salary employees (19.4%).

**Table 3:** Choice of government scheme's benefit options

	Salary category (%)				
		
Benefit option chosen	Lower skilled	Skilled	Highly-skilled	Supervisory and Senior Management	Total
Option 1: Low cost	19.4	9.4	0.5	1.7	4.4
Option 2: Low Cost	13.9	7.3	0.5	0.0	3.2
Option 3: Mid-range savings	11.1	5.2	11.1	5.0	8.8
Option 4: Comprehensive	55.6	75.0	80.6	65.0	74.8
Option 5: Comprehensive	0.0	3.1	7.4	28.3	8.8
Total	100 (36)	100 (96)	100 (216)	100 (60)	100 (408)

### Factors affecting uptake of the government scheme

For the insured, the most important reasons for joining the government scheme across all salary categories were the affordability of member contributions (67.4%), perceptions that it had better benefits and covered more dependents (37.9%) (Table 4).

**Table 4:** Factors influencing the decision to join the subsidised government scheme

	Salary category (%)				
		
Factors Affordability	Lower skilled	Skilled	Highly-skilled	Supervisory and Senior Management	Overall(%)
Insured joined because scheme affordable	57.9	65.0	70.2	67.2	67.4
Uninsured did not join because scheme expensive	40.2	40.2	27.1	33.3	34.8
Uninsured would join if scheme more affordable	34.0	32.0	34.6	13.3	32.8
Benefit options and coverage					
Insured joined as scheme offered better benefits and covered more dependents	36.8	41.2	33.5	49.2	37.9
Uninsured would join if more dependents were covered	2.1	2.1	0.0	0.0	1.2
Administrative complexity of scheme					
Insured joined as administrative procedures easy	2.6	10.3	6.0	11.5	7.5
Uninsured did not join as administrative procedures complex	28.9	21.7	17.3	20.0	21.9
Information about scheme					
Uninsured did not join scheme as lacked information	23.7	23.7	15.8	6.7	19.9
Uninsured would join if more information provided	2.1	2.1	0.0	0.0	1.2
Need for health insurance					
Uninsured did not join as insurance not needed	10.3	15.5	17.3	26.7	15.2
Uninsured would join if there was a health need	41.2	44.3	31.6	33.3	38.0

Multiple-response questions.

Amongst the uninsured, 40.2% of those in the lower-salary categories (lower skilled and skilled) cited lack of affordability as a reason for not joining and almost a third of all the uninsured across the three lower-salary categories noted that they would join if the scheme was made more affordable. Among respondents, 28.9% of those in the lower skilled and 21.7 in the skilled categories stated perceived administrative complexities had deterred them from joining the scheme, while 23.7% of lower skilled and skilled) stated lack of information about the scheme as important obstacles to enrolment. Among the uninsured, 26.7% of those in the higher-salary grades, did not join because they believed they did not need health insurance. However, more than a third of them said they would join if they had a health need.

In order to assess affordability, we examined employees’ contribution as a percentage of the lowest monthly income for each income category. As noted earlier, the government scheme contributions are income-based, with the monthly contributions varying according to the employee's salary, choice of benefit option and number of dependents. Despite the subsidy for low-income employees, on average those in the lower-salary categories still paid a higher percentage of their salaries for health insurance than those with greater income (with the exception of the fully subsidised option) (Table 5). For example, in the low-cost option 2, the health insurance payment constitutes 7.6% of income for someone in the lowest salary category 1 who earns $475 per month, while this option is only 1.7% of monthly income for someone in category 5 who earns $6,000 per month.

**Table 5:** Employee contributions (percent of monthly salary) towards monthly insurance contributions

	Salary category				
	
Benefit option	Lower skilled	Skilled	Highly-skilled	Supervisory	Senior Management
Low-cost option 1	0.0	0.0	3.5	1.6	0.5
Low-cost option 2	7.6	6.5	4.1	2.7	0.9
Mid-range savings option 1	13.0	10.2	7.0	5.2	1.7
Comprehensive option 2	13.5	10.5	8.0	5.8	1.9
Comprehensive option 3	34.5	27.0	16.9	10.7	3.4

1US$=7.5 South African Rand.

Financial contributions calculations assume a family of three (member, and an adult and child dependant). Percent monthly contribution is calculated as a proportion on the lowest level in each salary category. Income from other household members is unknown, and hence not included in the calculation.

## Discussion

In 2003, prior to the government scheme, insurance coverage among South African civil servants was 56% (35). Our analysis shows that two years after the government scheme was initiated in 2006, 74.3% of civil servants were insured, and 41.9% of these belonged to the government scheme. Although evidence suggests that membership has increased, with 53.8% of civil servants enrolled in the government scheme in 2012 (27), other studies on enrolment in health insurance schemes in Ecuador, Ghana, Mali, Senegal and Uganda, have found similar low levels of enrolment (36–40).

The newly-insured group included those from population groups who commonly experience financial and other access barriers, such as younger employees, women, unmarried single people, black Africans, and those living in informal housing or with lower-incomes. Therefore, in contrast to private health insurance in the general population, where 71% of members are located in the richest 20% of the population (41), the government scheme is comparatively pro-poor. Nevertheless, a considerable portion of socio-economically vulnerable groups remained uninsured (more than half of the lowest salary category for example), including men, black African or coloured race groups,^7^ less educated and lower-income employees, and those living in informal housing. This is despite membership for the lowest salary tier being fully subsidised.

Factors discouraging or deterring enrolment included affordability, the perceived administrative complexity of joining the scheme, and difficulties in obtaining information about the benefit options. Moreover, the comparatively poorer uptake of the government scheme in the more rural North West and KwaZulu-Natal provinces, may reflect underlying variations in geographical access to services (Table 2). Proximity of a primary care provider contracted with the scheme is likely an important factor influencing a potential member's decision to join the scheme. Transport costs have been shown to be an important barrier to accessing care in the South African setting (42), and the distance to a scheme-contracted provider may increase problems of affordability. This was likely an important issue in some provinces at the time of the survey. As argued in a recent review of UC in Thailand, ‘Financing reform must go hand in hand with ensuring physical access to services.’ (7, p. 17). In 2009, to improve access to primary health care services, the government scheme in South Africa expanded the network of primary healthcare service providers and geo-mapped members’ homes and workplaces against the provider in order to improve availability (43). In 2010, the scheme reported reaching a target of having at least 90% of members within 10 km from the nearest network provider (43). At a provincial level, this target was achieved in four of the country's nine provinces (Free State, Gauteng, KwaZulu-Natal and the Western Cape); in the rural North West province 84.8% of members were within 10 km of a registered provider. It will be important to document whether these changes have diminished the differentials between membership across provinces.

Affordability (or lack thereof) of member contributions was an important factor encouraging (or discouraging) enrolment in the government scheme. As Carrin et al. observe (44, p. 803), ‘Affordability of premiums or contributions is often mentioned as one of the main determinants of membership.’ The South African Ministerial Task Team commissioned investigation of low-income medical schemes found that ‘… the fundamental obstacle to expanding coverage to low-income households in South Africa remains affordability’ (24, p. 124). Several other studies have pointed to premiums being unaffordable as a factor discouraging demand for insurance in West Africa (45), Kenya (46) and India (47). Of note, a similar scheme to that studied here was implemented in Botswana in 1990, with all government employees entitled to a 50% subsidy from the government for health insurance. Nearly 70,000 members had enrolled by 2010 (48).

Preferences and expectations of the range of services and approved providers within benefit options can encourage (or deter) enrolment. Earlier research among households in South Africa indicated dissatisfaction and poor perceptions of public health services (10, 24), creating a preference for private health care, including primary and inpatient care. This might explain the relatively poor uptake of low-cost option 1, despite a full subsidy for those in the lower-salary categories. The ‘free’ low-cost option only provides members with access to basic outpatient services at pre-specified facilities and public hospitals, which may conflict with their strong preference for private primary and inpatient care. This might also explain the popularity across all salary categories of comprehensive option 4, which provides access to any private hospital.

The study identified perceptions and understandings of insurance, particularly among low-income employees, as a barrier to enrolment in the government scheme. These point to peoples’ underlying understandings of the potential role that insurance might play in either reducing or averting health care costs. This suggests a need for effective communication strategies to enhance knowledge about concepts of insurance to encourage enrolment in a health insurance scheme. The findings also suggest that older people (i.e. 60 years and older), whites, those in higher salary categories and tertiary education who probably have been with their current scheme for a long time may have ‘brand loyalty’ and consumer inertia, even if the new scheme offers better value for money due to the subsidy. Further research could more clearly define reasons and preferences for this.

Previous research exploring low enrolment in a community health insurance (CHI) scheme in Uganda identified ‘a mixed understanding on the basic principles of CHI and on the routine functioning of the schemes’, lack of information, affordability, poor quality of care, enrolment complexities and issues of trust as barriers to enrolment (38, p. 172). Similarly, in Ghana, a household study of the National Health Insurance Scheme identified premiums, registration fees and administrative arrangements as key factors influencing enrolment and retention (49). In Uganda (38) and Tanzania (50), lack of familiarity with community insurance schemes, particularly insurance principles of pooling and prepayments, contributed to low levels of enrolment. However, as Basaza et al. (38, p. 182) caution, ‘… a good understanding of CHI principles, per se, will not directly translate into increased enrolment.’ Qualitative research can improve understanding of the ways in which quality of care, benefit options, contributions and information shape peoples’ knowledge and views of health insurance and their decision to enrol.

Being a cross-sectional survey of existing civil servants, the study was unable to examine the period prior to the government scheme (i.e. pre 1993 when enrolment in one of a few pre-determined schemes was mandatory for some employees, or the period 1993 to 2005, where employees were free to choose which scheme they joined). The cross-sectional design cannot examine the institutional context within which insurance for civil servants has operated, changes that occurred in the scheme and how these may impact on participation. The ability to draw conclusions is also limited by the timing of the survey, which was only about three years after introduction of the scheme. The frequent changes made to the scheme in the period preceding this study and thereafter, restrict our ability to compare the study findings with outcomes of schemes in other countries or contexts. Also, it is possible that factors influencing enrolment in the long-run vary from those described here in the relatively early stages of the scheme. Moreover, data on the influence of perceptions and experiences of public health services on the decision to join the government medical scheme was not collected.

## Conclusion

Introduction of low-cost options which are fully subsided resulted in an increase in membership among low-income public-sector employees. However, uptake of membership particularly by young, black African or coloured groups, men, lower-income employees, those with no, or only primary education and in rural provinces was sub-optimal, suggesting that barriers remain. Importantly, these are the same population groups that have limited access to care within the existing publicly funded system. Lower-income employees were found to contribute a higher percentage of their salaries towards health insurance than higher-income employees indicating inequity in the government insurance scheme. The findings suggest that financing reforms intended to move towards UC must also take into account geographical and administrative access. Improving quality of care within public facilities is critical for improving public perceptions and encouraging the uptake of insurance especially among low-income households. Moreover, reforms need to consider the benefit options carefully, and must pay attention to the choice and geographical location of providers.

## Footnotes

1 The lowest cost benefit option is fully subsidised for those in the two lowest salary categories; for the other four benefit options, the government pays 75% of the employee's total monthly contribution, subject to an upper-limit.

2 Employees appointed prior to 2006 still received a subsidy for membership of any insurance scheme.

3 Retired civil servants were not included in the study sample. This population was initially excluded from participation in the government scheme, but later included.

4 As brokers could not make any financial gains from enrolling civil servants in the government scheme, we considered them unlikely to have any role or influence on the decision to enrol. Hence they were not considered in this study.

5 1US$=7.5 South African Rand.

6 Networks refer to designated health-care providers contracted through the government scheme to provide services to members.

7 In this paper, racial categories structured through apartheid are used in recognition that race remains an important social and economic fault line in the post-apartheid context.
